# Analysis of H3K4me3 and H3K27me3 bivalent promotors in HER2+ breast cancer cell lines reveals variations depending on estrogen receptor status and significantly correlates with gene expression

**DOI:** 10.1186/s12920-020-00749-2

**Published:** 2020-07-03

**Authors:** Damien Kaukonen, Riina Kaukonen, Lélia Polit, Bryan T. Hennessy, Riikka Lund, Stephen F. Madden

**Affiliations:** 1 Data Science Centre, Royal College of Surgeons in Ireland, Dublin, Ireland; 2 Turku Bioscience, University of Turku and Åbo Akademi University, Turku, Finland; 3 Institute Cochin, University Paris Descartes, Paris, France; 4 Medical Oncology Group, Department of Molecular Medicine, Royal College of Surgeons in Ireland, Dublin, Ireland

**Keywords:** Breast Cancer, HER2 +, Epigenetic modifications, Histone trimethylations, Bivalency, Gene expression, ChIP-seq, GRO-seq, RNA-seq

## Abstract

**Background:**

The role of histone modifications is poorly characterized in breast cancer, especially within the major subtypes. While epigenetic modifications may enhance the adaptability of a cell to both therapy and the surrounding environment, the mechanisms by which this is accomplished remains unclear. In this study we focus on the HER2 subtype and investigate two histone trimethylations that occur on the histone 3; the trimethylation located at lysine 4 (H3K4me3) found in active promoters and the trimethylation located at lysine 27 (H3K27me3) that correlates with gene repression. A bivalency state is the result of the co-presence of these two marks at the same promoter.

**Methods:**

In this study we investigated the relationship between these histone modifications in promoter regions and their proximal gene expression in HER2+ breast cancer cell lines. In addition, we assessed these patterns with respect to the presence or absence of the estrogen receptor (ER). To do this, we utilized ChIP-seq and matching RNA-seq from publicly available data for the AU565, SKBR3, MB361 and UACC812 cell lines. In order to visualize these relationships, we used KEGG pathway enrichment analysis, and Kaplan-Meyer plots.

**Results:**

We found that the correlation between the three types of promoter trimethylation statuses (H3K4me3, H3K27me3 or both) and the expression of the proximal genes was highly significant overall, while roughly a third of all genes are regulated by this phenomenon. We also show that there are several pathways related to cancer progression and invasion that are associated with the bivalent status of the gene promoters, and that there are specific differences between ER+ and ER- HER2+ breast cancer cell lines. These specific differences that are differentially trimethylated are also shown to be differentially expressed in patient samples. One of these genes, HIF1AN, significantly correlates with patient outcome.

**Conclusions:**

This study highlights the importance of looking at epigenetic markings at a subtype specific level by characterizing the relationship between the bivalent promoters and gene expression. This provides a deeper insight into a mechanism that could lead to future targets for treatment and prognosis, along with oncogenesis and response to therapy of HER2+ breast cancer patients.

## Background

The Human Epidermal growth factor Receptor 2 (HER2) is enriched in ~ 20%, and the Estrogen Receptor (ER) is overexpressed in ~ 70% of all breast cancers. While both breast cancer subtypes defined by these receptors have been extensively characterized, the impact of ER within the HER2 subtype remains poorly understood. Specifically, what influence does ER overexpression have on epigenetic patterns, such as histone modifications in HER2+ breast cancer?

Histone proteins help define chromatin structure and can undergo a variety of post-translational modifications, such as acetylation, methylation, phosphorylation, and ubiquitination. These modifications can alter the chromatin folding and protein-chromatin interactions, leading to an impact on gene expression [1]. The modifications on the various histone residues have been implicated in cell differentiation as a response to environmental changes [1]. Modifications to histone residues are carried out by enzymes such as histone deacetylases, demethylases, and methyltransferases [2, 3]. Some histone modifications can be mutually exclusive on a specific residue, such as acetylation and methylation [4]. Among these post-translational modifications, histone mono- (me1), di- (me2), and tri-methylation (me3), and the dysregulation of histone lysine demethylases have been associated with cancer [5]. This presents a mechanism of cell regulation that warrants further study [1, 5].

There are two tri-methylations on histone three, located at lysine residues four (H3K4me3) and 27 (H3K27me3), that come together to form a phenomenon known as bivalency. H3K4me3 is a post-translational modification that occurs at the promoter region and is associated with the activation of nearby gene expression, whereas H3K27me3 is enriched in the inactive gene promoters [6]. Bivalent promoters are promoters where both marks are present, and commonly occurs in stem cells, especially embryonic stem cells. In this state the genes are poised to become either activated or repressed as the cell becomes more committed [6]. Previous studies have found stem cells in the tumour microenvironment, referred to as cancer stem cells, and have shown that they can impact tumour growth, invasion, and response to therapy [7]. This has led to studies implying that bivalency is occurring in cancer cells, and is involved in oncogenesis [8].

While one study looked into the bivalent promoter patterns between an ER+, normal, and embryonic stem cell lines [8], and another study looked into the patterns between the ER+, HER2+ and triple negative cell lines [9], no one has looked into the HER2 subtype specifically to identify the differences that the ER may bring on HER2+ cell lines within the context of bivalency. Clinically, HER2+/ER+ tend to have a worse prognosis than HER2+/ER- patients, therefore, in this study we aimed to assess differences found within the HER2 subtype by characterizing what impact the presence or absence of the ER may have on the bivalent promoters within the HER2+ breast cancer cell lines. To broaden our understanding of the bivalency phenomena, we also examined two key pathways (HER2 and ER) which are extensively studied and clinically relevant in breast cancer. We then looked at the status of downstream targets from these pathways that are indicative of pathway alteration. From here, we bring the study back to the clinical setting by taking identified candidate genes and characterizing their clinical relevance by significantly segregating patient survival groups based on gene expression levels. Furthermore, while previous studies have looked at gene expression levels in relation to histone modifications [9, 10], we assessed this relationship in our four HER2+ breast cancer cell lines; two HER2+/ER+ cell lines (MB361 and UACC815) and two HER2+/ER- cell lines (AU565 and SKBR3). This was validated using Global Run-on sequencing (GRO-seq) and allowed us to correlate our cell line data with patient expression and clinical outcome data. This allowed us to make more robust conclusions from our inference of the relationship between bivalency status and gene expression between the cell lines and breast cancer patient data.

## Methods

### Public data

ChIP-seq files for the H3K4me3 and H3K27me3 histone modifications for our 4 cell lines (AU565, MDA-MB-361, SKBR3, and UACC812) were downloaded from SRA, accession number GSE85158 [10]. From the same study (GSE96867), the corresponding RNA-seq files for all 4 cell lines were downloaded (accession number: GSE96860) [10]. There were two biological replicates for each ChIP-seq condition, and four RNA-seq replicates for each cell line. Full information about the cell line and SRA accession numbers can be found in Supplementary Table 1. Additionally, for validation we utilized GRO-seq which can be accessed using GSE96859 [10]. RNA-seq information from patient tumours were retrieved from The Cancer Genome Atlas (TCGA). Only patients from TCGA that were listed as HER2+ according to previous studies were used (Supplementary Table 2) [11]. Kaplan-Meyer RFS plots were made using KM Plotter [12].

### ChIP-seq workflow

Following Encode Guidelines, the subsequent workflow was used to process the ChIP-seq files downloaded from SRA [13, 14]. All software was run using the default settings or settings as suggested within their manuals. Any deviations will be mentioned. First, the files were converted to fastq format using SRAtools (version 2.8.1) [15] and then quality control was performed using FASTQC [16] with trimming done using Trimmomatic (version 0.38) [17]. Alignment was achieved using bowtie2 (version 2.3.2) [18, 19] to the Hg38 reference genome [20], with unaligned reads discarded. Samtools [21] was used to remove reads with a q score of less than 20 and to sort the reads prior to marking with duplicates using Picard (REMOVE_DUPLICATES = F, VALIDATION_STRINGENCY = LENIENT) [22]. Peaks were then called using HMCan (version 1.16) [23], with the merge distance set to 200 bp for the H3K4me3 reads and 3000 bp for the H3K27me3 reads. The wig files generated were converted to BigWig files using WigtoBigWig [24], and then were viewed using the integrative genomics viewer from the Broad Instituted (IGV) [25]. By visualizing the outputs of HMCan on IGV, we determine which replicates should be discarded by evaluating the fit between the peak called and the density of the signal. The remaining replicates had their BAM files merged using Samtools, and the peaks of the merged files were counted using HMCan. These final files were annotated to genes within − 2000 to 1000 base pairs (bp) from the UCSC annotated transcription start site (TSS) using ChIPseeker [26] and passed on for downstream analysis.

### RNA-Seq and GRO-seq workflow

Utilizing a previously published approach [27, 28], both the RNA-seq and GRO-seq workflow was as follows, with all software ran using the default settings or settings as suggested within their manuals. The files were downloaded from SRA and converted to fastq format using SRAtools [15]. Then quality control was done using FASTQC [16] and bbduk (https://jgi.doe.gov/data-and-tools/bbtools/), with alignment to the Hg38 reference [29] genome using HiSAT2 (version 2.1.0) aligner. The resulting gsnap.sam files where then converted to bam files and sorted based on coordinates using Samtools [21]. This was followed up with Stringtie for transcript assembly and BallGown to normalize and organize the read counts in FPKM or python to create raw read tables [28, 30].

### Visualization of histone modification in relation to gene expression

The average density of histone modification signal around TSS was visualized according to gene expression. Genes were separated into three categories using kmeans clustering on the RNA-seq data: high expression, medium expression, and low expression. Then the output of HMCan was used to compute the average density of the histone modification signal per bin (50 bp) around the TSS (−4kB, +4kB) for the three groups of expression.

### KEGG pathway analysis

To perform KEGG pathway enrichment analysis, the package clusterprofiler [31] was used. The *p*-value was adjusted using the Benjamini-Hochberg method [32], and an adjusted *p*-value of 0.05 was considered significant. The first analysis was done on genes that had either the H3K4me3 or H3K27me3 histone modification within − 2000 to 1000 bp from the TSS. Then the analysis was performed again but only on genes which had both marks within the same region. Both analyses were visualized using dot plots.

### Statistical analysis

A Kruskal-Walis test followed by a post-hoc Wilcoxon rank sum test of each possible pairwise comparisons were performed, comparing the gene expression levels of the three groups (H3K4me3, H3K27me3, or both) to determine if the distribution between them was significantly different. These distributions for each cell line were then visualized using box plots with Log2(x + 1) transformed gene expression values.

To look at the relationships on a pathway specific level, two gene lists were obtained from the KEGG data base, the HER (ErbB) signalling pathway (ko04012) and the Estrogen Signalling Pathway (ko04915), and subsetted into their respective pathways from our dataset [33]. From these subsets of genes, a Fisher Exact test was used to compare the ratios of H3K4me3, H3K27me3 or both as conditions in each pathway with the total distribution of the three conditions in the entire dataset, as well as comparing the proportion of differentially bivalent genes found in the downstream targets to the global distribution. A Kruskal-Wallace test followed by a Wilcoxon rank sum post-hoc test for each possible pairwise comparison was also performed to compare the mark distribution with their respective gene expression levels within the cell lines. A student’s T-Test was used to compare the GRO-seq expression levels of key genes found in the HER and Estrogen signalling pathway between the ER+/HER2+ and ER−/HER2+ cell lines. The *p*-values were adjusted using the Benjamini-Hochberg [32] and those comparisons with an adjusted *p*-value of less than 0.05 were considered significant.

### TCGA analysis

To correlate the histone modification profiles with patient tumours, RNA-seq data was downloaded from both TCGA. For TCGA, the package TCGAbiolinks [34] in R was used, and only information from patients that were HER2+ was included. Differential expression analysis was performed using DESeq2 [35] on the gene signatures that were identified as regulated by the HER2 and ER pathways in previously published work (Supplementary Table 3) [36].

## Results

### Bivalency in HER2+ cells

**Fig. 1:**
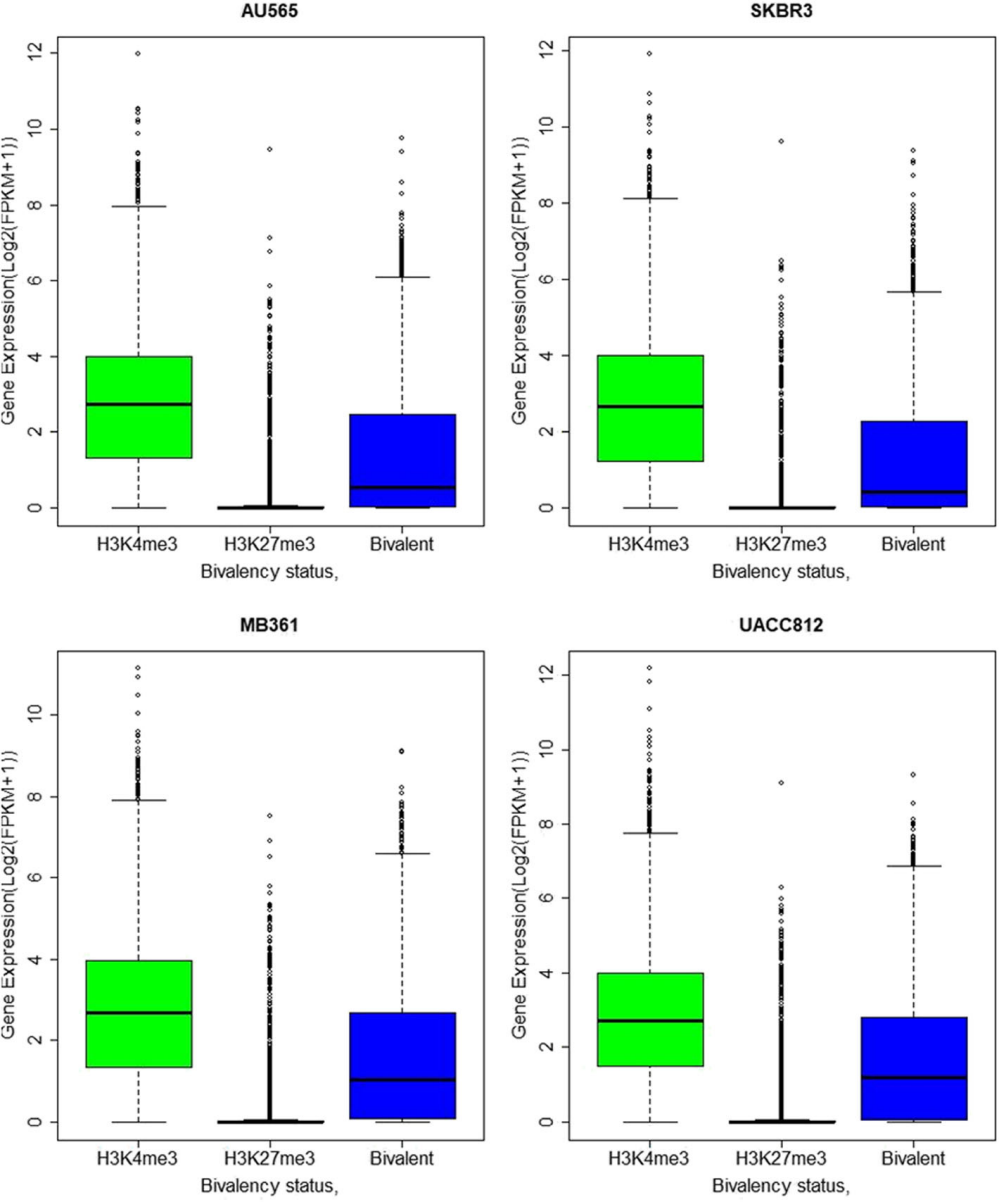
The bivalent promoter status correlates significantly with gene expression. The box plots visualizing the Log2(x + 1) distribution of gene expression from the 3 bivalent promotor states; H3K4me3 marked, H3K27me3 marked, or both. The top two plots are of ER- cell lines, the bottom two are ER+

Our objective was to characterize the relationship between the H3K4 and H3K27 trimethylations with gene expression to confirm that these marks retained their previously identified impact within our cell lines. In this instance, we considered a mark to be near the transcription start site (TSS) if it was within − 2000 bp to 1000 bp from the TSS. Using ChIP-seq data combined with the RNA-seq data, we confirmed that among all the cell lines, genes with a H3K4me3 mark near the TSS have higher expression, those with an H3K27me3 mark have low expression, and those with both marks have a distribution of expression levels somewhere in between (Fig. 1). A post-hoc Wilcoxson test confirms that this trend is significant (*p*-value < 0.05) for all relationships across all the cell lines. Despite this significant relationship, there appears to be a substantial number of outliers in each group.

**Fig. 2:**
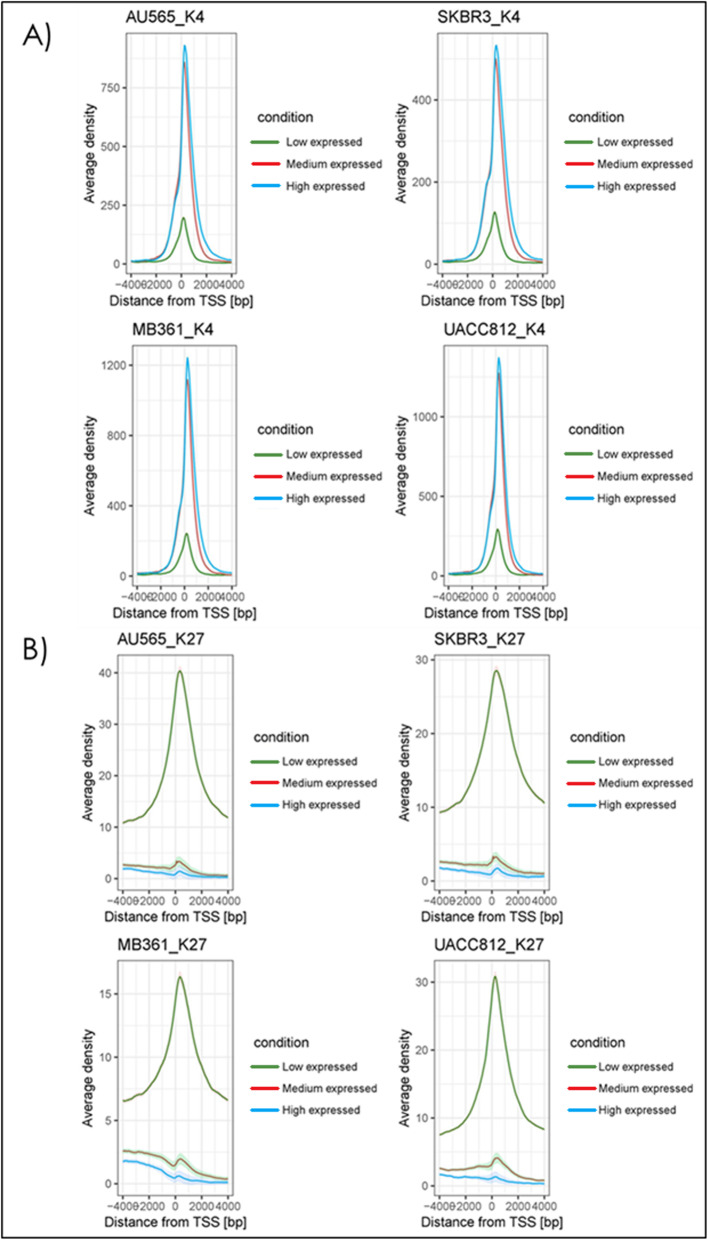
The average density of histone modification signal around TSS according to gene expression. The visualization of the histone modification location and gene expression correlation plots for the H3K4me3 (**a**) and H3K27me3 (**b**) marks in the 4 HER2+ breast cancer cell lines. For each plot, the gene expression was clustered into three groups (Low, Medium and High expression) using kmeans, and each cluster was plotted according to the average density of the peak with respect to distance from the transcription start site (TSS) for their corresponding genes

Next, we visualized the relationship between the different bivalent histone modifications and gene expression by plotting peak density with respect to distance from the TSS across three different clusters of gene expression levels (high, medium, and low) (Fig. 2). As expected, higher peak density of the H3K4me3 near the TSS is correlated with the high and medium gene expression clusters, and the opposite is the case for the H3K27me3. These plots also show that the histone modification has to be located near or on the TSS to have the expected impact on gene expression.

### HER2+/ER+ and HER2+/ER- cell lines have different bivalently marked pathways

**Fig. 3:**
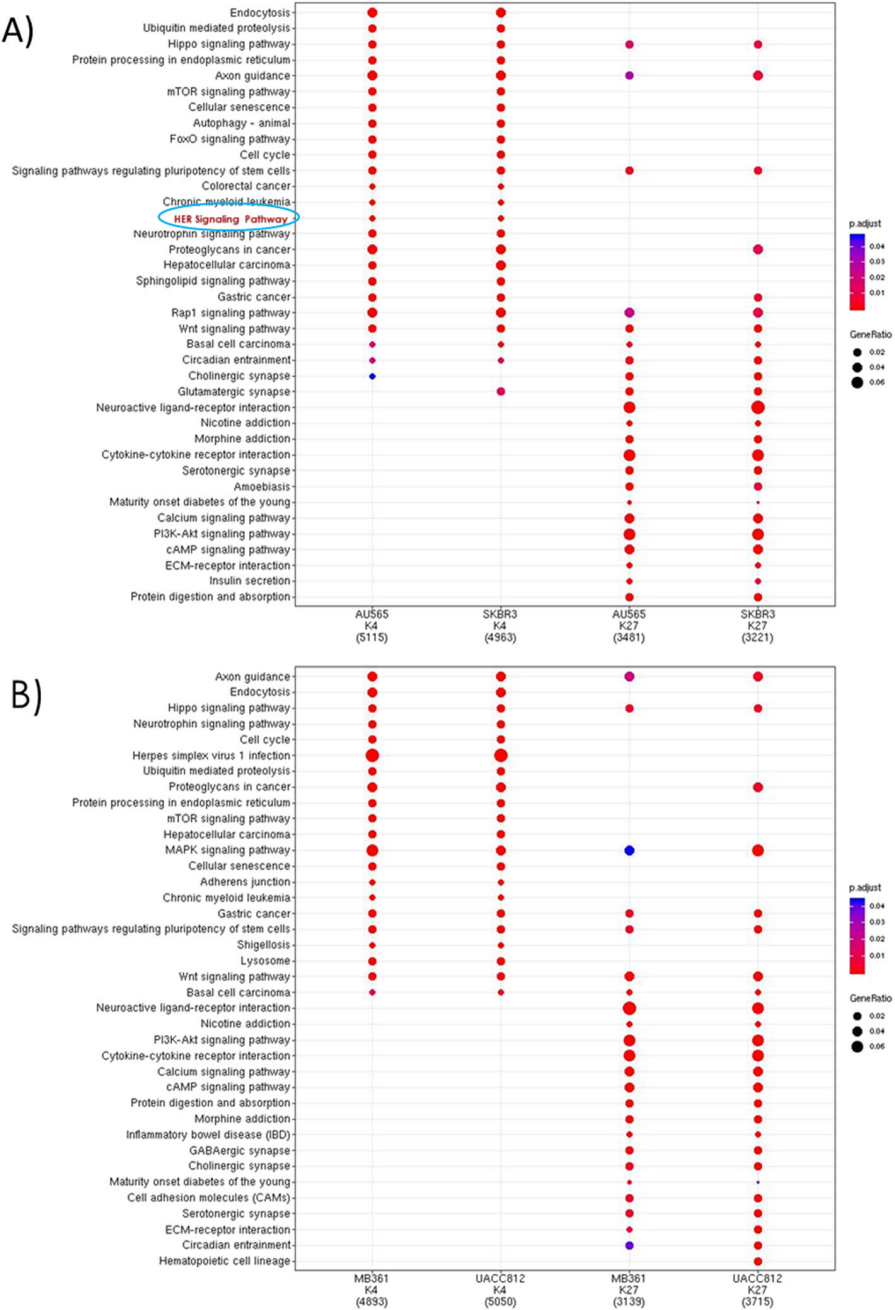
KEGG pathway enrichment shows differences between ER+ and ER- HER2+ cell lines. The KEGG pathway enrichment analysis for the genes containing the H3K4me3 (K4) or H3K27me3 (K27) histone modifications for the ER- (**a**) and ER+ (**b**) HER2+ cell lines used in this study. The number in brackets at the bottom represents the number of individual genes with peaks located within −2000 to + 1000 bp from a TSS, the size of the dot represents the gene ratio of unique genes found to contain a peak within that pathway, the pathways that are enriched are labeled on the left-hand side, and the colour of the dot represents the range of adjusted *p*-values (*p*-value < 0.05)

Next, we assessed the differences between HER2+/ER+ and HER2+/ER- cells lines on a pathway level. Figure 3 shows the KEGG pathway enrichment analysis looking at the distribution of both marks across enriched pathways. While the list of enriched pathways does not differ between the two groups, the order of the rankings differs, suggesting that there are differences on the gene level. Of note, we see several pathways related to cancer progression and metastasis as well as tumour suppression being regulated by bivalency. Interestingly, the HER signalling pathway (HER 1–4) is seen enriched in the HER2+/ER- cell lines, but not with the HER2+/ER+ cell lines. This demonstrates a greater reliance on the HER pathway in the HER2+/ER- cell lines than in the HER2+/ER+ cell lines.

We found three genes within the HER pathway (Supplementary Table 4) that appear differentially marked between HER2+/ER+ and HER2+/ER- cells lines: PAK5 (bivalent in ER- but is H3K27me3 marked in ER+), HBEGF (H3K4me3 marked in ER- and bivalent in ER+), and SHC4 (H3K4me3 marked in ER- and either bivalent or H3K27me3 marked in ER+). Remarkably, both SHC4 and HBEGF are found in the Estrogen signalling pathway (Supplementary Table 5), and the GRO-seq data shows that are statistically differentially expressed between the ER+/HER2+ and ER−/HER2- cell lines (HBEGF *p*-value = 3.74 × 10^− 2^ and SHC4 *p*-value = 4.46 × 10^− 2^). This would indicate that they both have a different role to play in each pathway, or, at the very least, in each group. SHC4 and PAK5 have both been studied in breast cancer, with the former being used as one of 12 gene signatures linking molecular mechanisms to disease prognosis [37], and the latter being associated with invasion, metastasis, and poor outcome in several cancers [38–40]. The corresponding Global run-on sequencing (GRO-seq) used to validate the gene expression levels can be found in Supplementary Table 6.

### Bivalency is an enriched phenomenon in the HER signalling pathway

When we looked at bivalency in HER and ER signalling pathways, we saw that the HER pathway is statistically significantly different from the background pattern, indicating that bivalency is an important regulatory mechanism for HER signalling. However, the Estrogen signalling pathway is trending towards being significantly different in the HER2+/ER+ cell lines but not in the HER2+/ER- (Table 1). This shows that the HER pathway is important in all 4 HER2+ cell lines, but the ER pathway is only regulated differently from the background in HER2+/ER+ cell lines.

**Table 1:** Adjusted *p*-values for the Fisher exact test to compare the distribution of the 3 bivalent promotor statuses (H3K4me3, H3K27me3, or both) against the background

	ER- Cell Lines		ER+ Cell lines	
	AU565	SKBR3	MB361	UACC812
HER Pathway	5.07E-03	2.19E-03	4.22E-03	2.47E-03
Estrogen Pathway	0.53	0.46	0.09	1.76E-03

### Differentially bivalently marked genes and pathways identified between HER2+/ER+ and HER2+/ER- cells

**Fig. 4:**
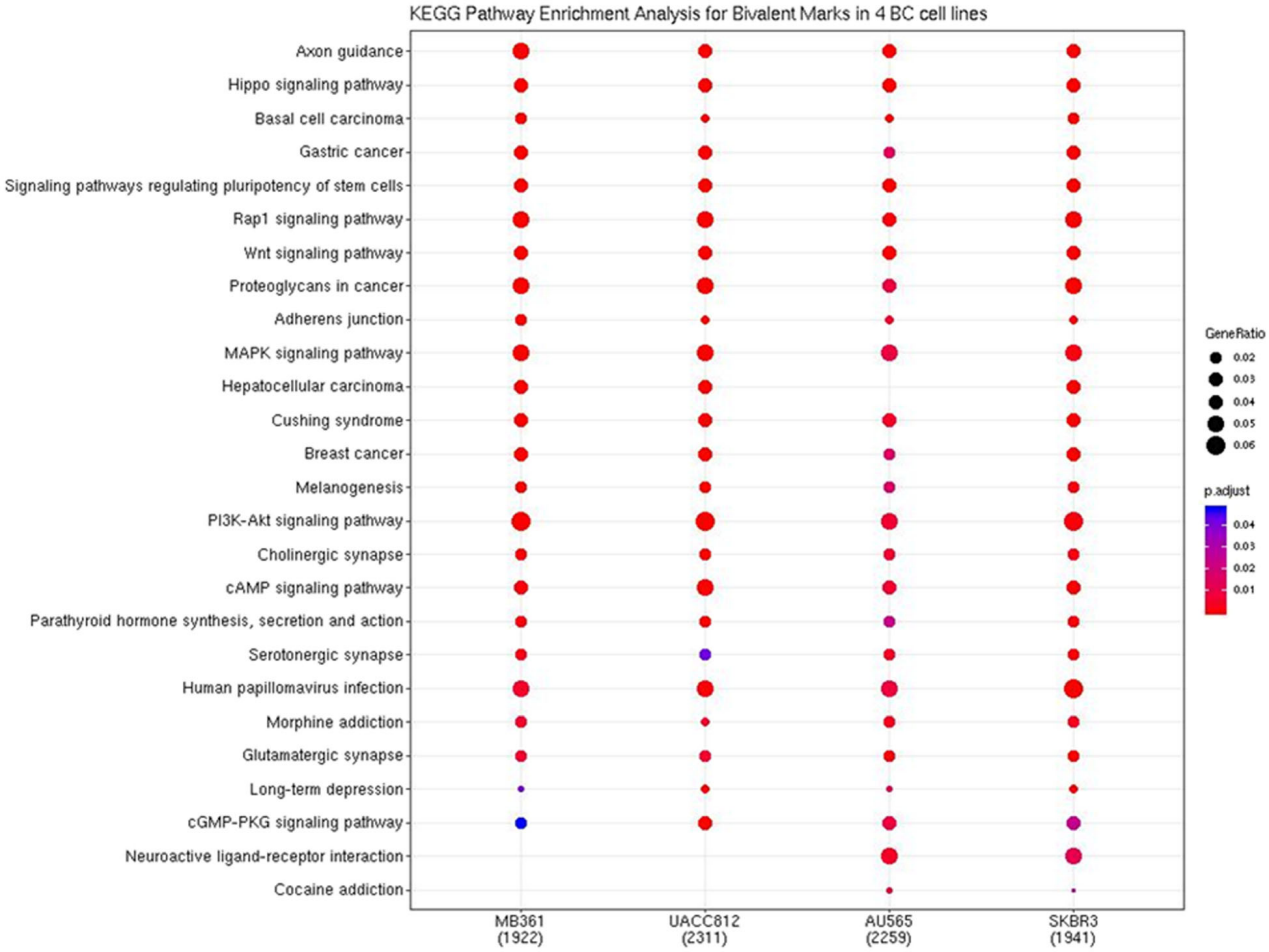
Enrichment analysis of genes with both marks shows only one difference between ER+ and ER-. Dot plot of the KEGG Pathway enrichment analysis for the genes that have both an H3K4me3 and H3K27me3 histone modification within -2000 to 1000 bp from the TSS for 4 HER2+ breast cancer cell lines. The number of genes with both modifications are located in the bottom brackets, the enriched pathways are labeled on the left-hand side, and gene ratio and adjusted p-vales are determined by dot size and colour respectively

Looking at the pathway level, we see that there are few differences between the cell lines in terms of bivalency status (Fig. 4). The pathway that really stands out as a difference between HER2+/ER+ and HER2+/ER- is the Neuroactive ligand-receptor interaction pathway.

Despite the fact that not many pathways that are differentially bivalent between the ER+ and ER- cell lines, there are several genes that are. Of the 57,865 annotated genes (both coding and non-coding) found in the Hg38 reference genome, 19,914 were found to have one or both marks in at least one of the four cell lines. Of this, 545 genes were shown to be bivalent in HER2+/ER- and not in HER2+/ER+, and 466 vice versa. Additionally, it does show that the presence of a bivalent mark is uncommon overall, suggesting that those pathways which have a high ratio of genes with a proximal bivalent mark are important.

### HER2+ breast cancer patient gene expression patterns correlates with HER2+ cell line bivalency data

One of the ways to assess the impact of epigenetic regulation on the expression of a pathway is to evaluate the expression of targets downstream from the pathway. A previous study [41] generated a list of genes that directly correlate with the activation or deactivation of several important pathways found in breast cancer, including the ER and HER2 pathways. For this study, we did a differential expression analysis of these genes signatures in HER2+ patients from the Cancer Genome Atlas (TCGA) dataset, and then correlated them with their bivalent markings in our cell lines.

While we found 32 of these genes differentially expressed between the HER2+/ER+ and HER2+/ER- patients, only one is differentially marked between our HER2+/ER+ and HER2+/ER- cell lines; Kynurenine 3-monooxygenase (KMO) (Table 2). KMO has been previously shown to promote breast cancer progression in triple negative breast cancer while being highly upregulated in HER2+ breast cancer [42]. It is interesting that here we show that there is differential gene expression among those patients who are HER2+, and that difference is not only correlating with ER status, but it also correlates with a difference in histone modification.

**Table 2:** The significant genes from the differential expression analysis of downstream targets to the HER2 pathway, with the trimethylation mark status shown for the four cell lines used in this study

Genes	TCGA Mean ER-	TCGA Mean ER+	Adjusted ***p***-value	AU565	SKBR3	MB361	UACC812
EGFR	4255.32	1032.16	5.14E-07	Both	Both	Both	Both
HDAC11	1178.71	2209.11	5.14E-07	H3K4	H3K4	H3K4	H3K4
PADI2	13,566.18	3909.07	9.85E-07	H3K4	H3K4	H3K4	H3K4
MSMB	108.29	1019.47	7.81E-06	H3K4	H3K4	H3K27	NA
SPRY4	3322.68	2033.51	1.03E-05	H3K4	H3K4	H3K4	Both
PRKACB	3114.35	7224.62	3.13E-05	Both	H3K4	H3K4	H3K4
C2orf54	6664.59	1740.34	8.39E-05	H3K4	H3K4	Both	H3K4
COL9A2	530.53	1131.71	1.46E-04	Both	Both	H3K4	Both
TJP3	1814.35	2869.42	2.37E-04	H3K4	H3K4	H3K4	H3K4
DSC2	6958.56	3751.01	8.48E-04	H3K4	H3K4	H3K4	H3K4
KRT7	46,998.21	21,373.81	8.48E-04	H3K4	H3K4	H3K4	H3K4
MICALL2	798.74	1120.52	8.48E-04	Both	Both	Both	Both
GGT1	4120.00	1856.94	1.00E-03	H3K4	NA	H3K4	NA
HLA-DOB	283.85	146.84	1.00E-03	NA	H3K27	H3K27	H3K27
TES	6267.21	4322.84	1.00E-3	H3K4	H3K4	H3K4	Both
GRB7	19,522.94	9486.44	1.04E-03	H3K4	H3K4	H3K4	H3K4
TMPRSS2	2215.91	1031.48	1.40E-03	H3K4	H3K4	H3K4	Both
BCL11A	431.03	161.07	1.40E-03	Both	Both	Both	Both
KMO	2436.18	1157.30	1.40E-03	H3K4	H3K4	Both	Both
CDR2L	3813.56	2289.99	1.73E-03	H3K4	H3K4	H3K4	H3K4
MACROD1	415.71	582.91	2.08E-03	H3K4	Both	Both	Both
STARD3	21,362.24	12,410.42	3.81E-03	H3K4	H3K4	H3K4	H3K4
HER2	302,441.26	168,929.72	5.10E-03	H3K4	H3K4	H3K4	H3K4
VLDLR	1718.06	1004.34	7.08E-03	NA	NA	NA	NA
RNF123	1765.68	2124.94	0.01	NA	NA	NA	NA
PKD1	3058.32	3757.13	0.02	NA	NA	NA	H3K4
EGR1	9793.62	18,680.44	0.03	Both	Both	Both	Both
NR1H2	2741.91	3140.22	0.03	H3K4	Both	H3K4	H3K4
ST6GAL1	8994.97	5555.26	0.03	Both	H3K4	Both	Both
ATG2A	1652.56	2460.58	0.03	H3K4	H3K4	H3K4	H3K4
ITPKC	2043.29	2397.91	0.04	H3K4	NA	NA	NA
CEACAM7	182.03	97.02	0.05	NA	H3K27	H3K27	NA

Both referring to having both the H3K4me3 and H3K27me3 marks

When we applied the same analysis for the downstream targets of the ER, we see a slightly different pattern than what we saw in the HER2 targets (Table 3). For one, the majority of the pathway appears to be H3K4me3 or bivalently marked for all four cell lines, irrespective of ER status. This pattern is something that we would have expected to more likely occur in our HER2 downstream patterns, not the ER, as these are all HER2 enriched cell lines. Even so, there are three genes that are both differentially expressed and differentially marked between the HER2+/ER+ and HER2+/ER- subgroups: estorgen receptor (*ESR1*), Trefoil factor 3 (*TFF3*), and Hypoxia inducible factor alpha inhibitor (*HIF1AN*).

**Table 3:** The differential expression analysis for the genes that are downstream targets of the ER pathway with the trimethylation mark status shown for the four cell lines used in this study

Genes	TCGA Mean ER-	TCGA Mean ER+	Adjusted *p*-value	AU565	SKBR3	MB361	UACC812
ESR1	1957.85	28,587.25	4.97E-26	Both	Both	H3K4	H3K4
TTLL4	2292.15	1222.79	5.87E-14	H3K4	H3K4	H3K4	H3K4
CA12	6494.32	26,181.72	2.39E-11	Both	H3K4	H3K4	Both
GATA3	10,042.38	30,905.96	1.78E-09	Both	Both	Both	Both
IDE	2696.24	2470.56	5.06E-06	H3K4	H3K4	H3K4	H3K4
TIMM17B	2089.91	1922.64	7.35E-06	NA	NA	NA	NA
RAB11A	13,538.06	12,663.28	1.68E-05	H3K4	H3K4	H3K4	H3K4
TFF3	11,984.35	16,748.75	4.28E-04	H3K27	H3K27	H3K4	H3K4
CYB561	20,940.65	17,868.85	5.41E-04	H3K4	Both	H3K4	H3K4
ST3GAL6	324.15	249.91	5.54E-04	NA	NA	NA	H3K27
FBP1	2520.97	5160.04	7.95E-04	H3K4	H3K4	H3K4	H3K4
LIN7A	116.18	352.67	7.53E-03	Both	Both	H3K4	Both
SOX13	4161.82	4182.33	9.74E-03	H3K4	H3K4	H3K4	H3K4
KIAA1279	2414.85	2747.07	0.01	NA	NA	NA	NA
C10orf116	3067.85	6545.65	0.01	NA	NA	NA	NA
TGFB3	4358.59	4501.40	0.01	H3K4	H3K4	H3K4	H3K4
HIF1AN	3677.74	4135.13	0.01	H3K4	H3K4	Both	Both
ANXA9	1033.79	2243.66	0.02	H3K4	NA	H3K4	H3K4
PCBP2	25,977.29	30,070.89	0.02	H3K4	H3K4	H3K4	H3K4

Both referring to having both the H3K4me3 and H3K27me3 marks

**Fig. 5:**
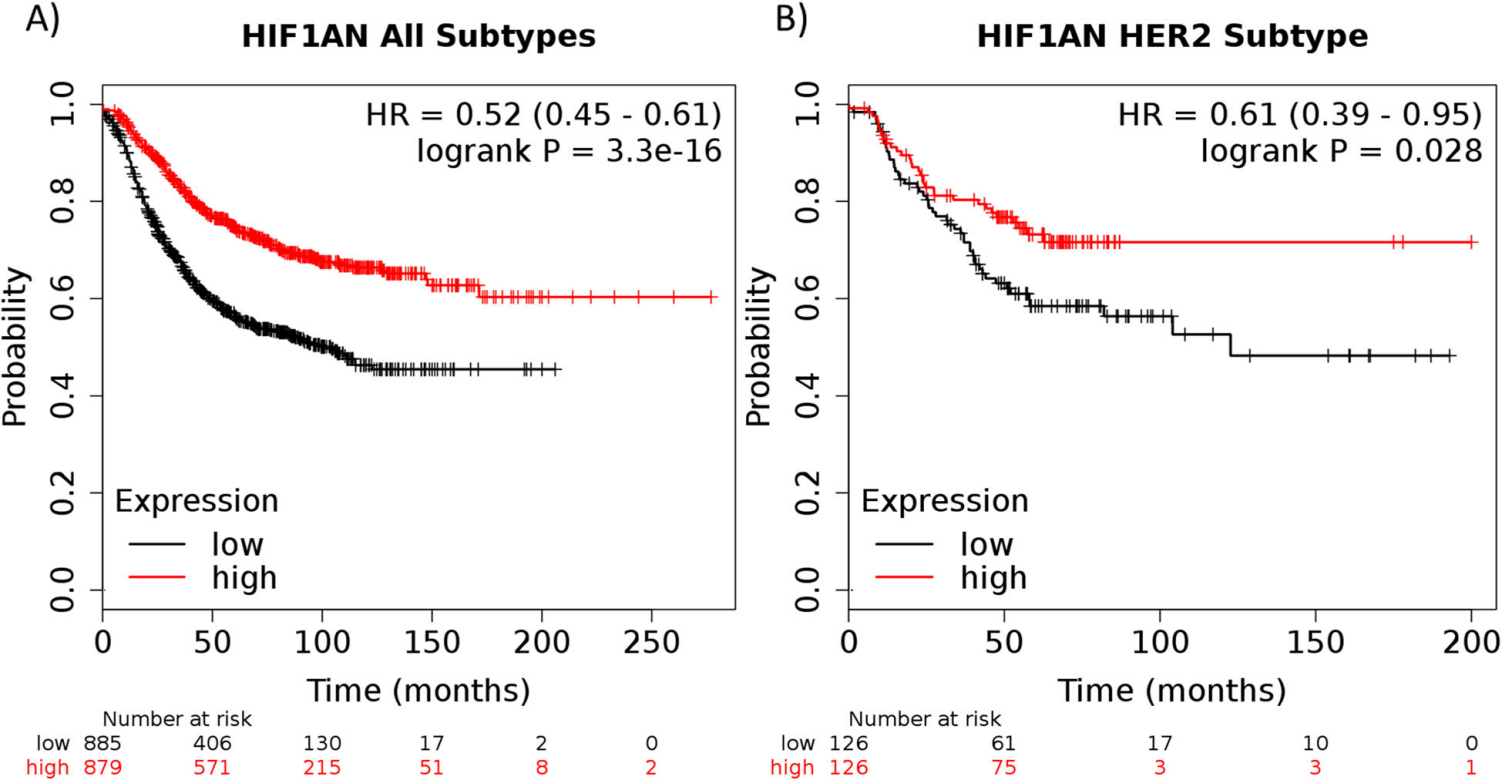
The Kaplan-Meyer RFS survival curves for all subtypes (**a**) and within the HER2 subtype (**b**), with the median value being the cutoff between high (red) and low (black) expression. The logrank *p*-values and hazard ratios can be found in the top left corner of each plot

While the *ESR1* was expected to be differentially expressed between ER+ and ER- subgroups, it is interesting that it is bivalent in ER- and H3K4me3 marked in ER+. This means that *ESR1* is constitutively active in ER+ cell lines while still being open to activation in ER- cell lines. We also observed that it was bivalent in two normal cell lines (data not shown), which raises the question as to what came first; the histone modification or the over expression of the *ESR1*. *TFF3* is also fascinating, as it has been seen with elevated levels in the blood in metastatic breast, and shown as a predisposition for invasion, and acts as a biomarker for endocrine response [43–45]. Additionally, one study has shown that low expression of HIF1AN led to an advantage for stem cells under hypoxic conditions [46], and another study showed that low activity of HIF1AN due to hypoxia was associated with metastasis in ovarian cancer through interactions with histone lysine methyltransferases [47]. In breast cancer, HIF1AN expression has been shown to be elevated in metastatic cases [48]. We can also see that HIF1AN has clinical relevance as there is significant correlation between high and low expression levels and patient outcome, in all subtypes as well as within the HER2 subtype (Fig. 5). In both instances, high expression of HIF1AN correlates with poor clinical outcome. Additionally, the number of genes that are differentially marked in these groups are significantly different than what we find in the global level for both the HER2 (*p*-value = 3.82 × 10^− 6^) and the ER (*p*-value = 7.3 × 10^− 3^) downstream targets. These results show the importance of studying bivalency status and pathway regulation, both on a gene level and as downstream targets, in breast cancer. Complete tables for downstream targets of the HER2 and estrogen receptor pathways can be found in Supplementary Table 7 and Supplementary Table 8.

## Discussion

In this study, we aimed to characterize the relationship between the bivalency marks (H3K4me3 and H3K27me3) and gene expression, as well as how the pattern changes in the presence or absence of the ER in HER2+ breast cancer. Our results show that there is a significant correlation between the mark status, the mark location, and gene expression overall, and that there are notable differences between the two subgroups (the HER2+/ER+ and HER2+/ER- cell lines). From here we can infer the correlation for several of the gene signatures that we looked at in the patient data with the corresponding bivalent mark status in our cell lines. We also show that several of the bivalent-regulated genes do have clinical significance. Overall, the study characterized the relationship in a way that, to the best of our knowledge, has not be done previously in HER2+ breast cancer data.

One of the more striking observations we made was that at a global level, only about one third of all genes have one or both of the bivalent marks. However, the two key breast cancer pathways we looked at, the HER and estrogen signalling pathways, were almost entirely marked, and still had a significantly different distribution from the general marking patterns. This highlights the importance of this phenomenon in cancer generally and HER2+ breast cancer specifically, as dysregulation of this bivalent process would clearly affect these key breast cancer pathways. Strikingly, we did see that the estrogen signalling pathway was only significantly different in the ER+ cell lines from the background, showing a difference, despite both being HER2 + .

While the estrogen signalling pathway was significantly different from the background in terms of mark distribution for the HER2+/ER+ cell lines, the HER signalling pathway was only shown to be significantly enriched for the HER2+/ER- cell lines in our KEGG analysis. When we looked at the gene level of the HER pathway, we saw that one of the differentially marked genes between the two groups was *HBEGF* (H3K4me3 marked in ER- and bivalent in ER+). While it has been shown to promote metastasis and macrophage-independent invasion [49], and act as an effective target for antibody bound nanoparticles for drug delivery in triple negative breast cancer [50], *HBEGF* is interesting due to its relationship with the Epidermal Growth Factor (EGF). *HBEGF* has been previously associated with EGF receptor and HER2 and it has a higher affinity for the EGF receptor than EGF itself [51]. Furthermore, we also saw that *KMO* both differentially regulated and marked in our analysis that looked at the downstream markers for the HER2 pathway in the HER2+ TCGA patients. *KMO* has been shown to have increased activity in breast cancer compared to normal cells, specifically being upregulated on the protein level in the HER2+ subtype and having elevated transcription levels in the Triple Negative subtype [52, 53]. *KMO* also has relatively lower levels of expression in the Luminal (ER+) subtype compared to the aforementioned subtypes [52, 53]. Unfortunately, the relationship between the ER and KMO remains uncharacterized and warrants further investigation as the presence or absence of the ER seems to correlate with *KMO* expression, even within the HER2 dominated subtype.

One of the other questions raised in this study is that, despite the strong significant correlation between mark status and gene expression levels, we see there are several genes that are outliers, specifically in the H3K27me3 group. One of the possibilities is that the H3K27me3 mark is either not on all of the locus within the cell, or that not all of the cells in our samples have the mark on gene all the time. While it is possible that if this phenomenon is dynamic enough to change for a few select genes within the cell cycle, it would seem more likely that the locus is differentially marked, given the gene expression is still relatively high for the cells.

While this study is not without its limitations, we were still able to accomplish our objectives; characterizing the phenomenon of bivalency and how it differs between ER+ and ER- subtypes within HER2+ breast cancer. The inclusion of gene expression data not only confirms what has been previously studied, but it allowed us to provide a more robust conclusion when comparing the gene expression levels in patients and inferring their relationship with the bivalency status within our cell lines. We have clearly been able to show how strong the relationship between the mark status and gene expression and how this affects key breast cancer pathways. Several of the key genes we have identified have been studied within the context of breast cancer, and here we have put forth a method by which these key genes may be (dys)regulated. Previous studies have repeatedly demonstrated a correlation between gene expression and response to therapy [54, 55] as well as suggesting that histone modifications enable a cell to adapt to changes in the environment [1]. Therefore, this study laid the foundations of exploring this relationship by showing the strong correlation between mark status and gene expression and shows that bivalency correlates with the driving factors behind the breast cancer subtypes. This is demonstrated in the key differences within the HER2 subtype in the presence or absence of the ER. Future studies can aim to understand how dynamic the system is, if there are differences in which locus are marked, and better characterize the bivalency patterns within specific subtypes, such as the HER2 or triple negative subtypes. Lastly, further studies into bivalency within the subtypes and how it regulates genes could provide new targets for therapy, as several of the sought after and currently targeted molecules for therapy, including EGFR, Src, and HER4 [56–58], are all shown as bivalent in this study. Further characterization of this phenomenon can lead to a better understanding of how resistance to therapy is acquired, and advance our depiction of oncogenesis, particularly between the different subtypes of this family of diseases.

## Conclusions

We further characterize the bivalency phenomena by focusing on a specific breast cancer subtype, HER2, and finding differences both on the gene pathway scale and within the clinical environment. We do this by reaffirming the relationship between the H3K4me3 and H3K27me3 statuses with gene expression and expanding this by showing differences between cell lines within the HER2 subtype based on ER status. This adds to the current understanding about the bivalency phenomenon and its role in breast cancer by showing there are differences within a subtype which are influenced by the presence of another receptor; in this instance the estrogen receptor is having an impact on the HER2 subtype. The influence of bivalency on these two receptors was demonstrated by their pathways being highly regulated by these histone trimethylations and the impact it has on the expression of downstream targets. We also show that these differences between the HER2+/ER+ and HER2+/ER- appear to go beyond the cell lines and are represented in differential expression of downstream genes within patients. In these instances, these differences include genes that are already known to have a role in breast cancer. Accordingly, we conclude that is it important to study the bivalency phenomenon within the subtypes to identify key differences that can stratify the disease further. We also suggest that the bivalency phenomenon should be further characterized within patient samples as well as within the other subtypes and other cancers. Doing so may help us better stratify patients within the HER2 and other subtypes and give us a better understanding of oncogenesis and how the cells will respond to treatment.

## Supplementary information

**Additional file 1: Supplementary Table 1.** Information and accession numbers for ChIP-seq and RNA-seq of the 6 cell lines used in this study.

**Additional file 2: Supplementary Table 2.** The TCGA patients that are HER2+ with their ER status as determined from a previous study. (CSV 3 kb)

**Additional file 3: Supplementary Table 3.** The genes identified in a previous study as markers for regulation in the ER and HER2 pathways.

**Additional file 4: Supplementary Table 4.** The list of genes in the HER pathway according to KEGG, with their histone trimethylation status and corresponding gene expression.

**Additional file 5: Supplementary Table 5.** The list of genes in the Estrogen Signaling pathway according to KEGG, with their histone trimethylation status and corresponding gene expression (in FPKM).

**Additional file 6: Supplementary Table 6.** The combined list of genes for the HER and Estrogen signaling pathways according to KEGG, with their corresponding histone trimethylation status and GRO-seq values in FPKM.

**Additional file 7: Supplementary Table 7.** The complete differential expression analysis of genes downstream of the HER2 pathway, comparing the ER+ with the ER- HER2+ patients in the TCGA data, with the corresponding bivalent promoter status found in our 4 cell lines.

**Additional file 8: Supplementary Table 8.** The complete differential expression analysis of genes downstream of the estrogen signaling pathway, comparing the ER+ with the ER- HER2+ patients in the TCGA data, with the corresponding bivalent promoter status found in our 4 cell lines.

## Data Availability

The datasets generated and/or analysed during the current study are available in the Gene Sequence Archive repository, references GSE85158 (ChIP-seq), GSE96867 (RNA-seq) and GSE96859 (GRO-seq). The SRA numbers for the ChIP-seq and RNA-seq can also be found in Supplementary Table 1. The TCGA data was downloaded using the R package TCGAbiolinks, and the Hg38 reference genome was downloaded from the UCSC webpage. Accession numbers for the TCGA data can be found in Supplementary Table 2. ChIP-seq: https://www.ncbi.nlm.nih.gov/geo/query/acc.cgi?acc=GSE85158 RNA-seq: https://www.ncbi.nlm.nih.gov/geo/query/acc.cgi?acc=GSE96867 GRO-seq: https://www.ncbi.nlm.nih.gov/geo/query/acc.cgi?acc=GSE96859 TCGAbiolinks: https://bioconductor.org/packages/release/bioc/html/TCGAbiolinks.html Hg38: https://hgdownload.soe.ucsc.edu/downloads.html#human

## Abbreviations

ANXA9: Annexin A9
ATG2A: Autophagy Related 2A
BCL11A: B-Cell CLL/Lymphoma 11A
C2orf54: Chromosome 2 Open Reading Frame 54
CA12: Carbonic Anhydrase 12
CDR2L: Cerebellar Degeneration Related Protein 2 Like
CEACAM7: Carcinoembryonic Antigen Related Cell Adhesion Molecule 7
ChIP-seq: Chromatin Immunoprecipitation Followed By Sequencing
COL9A2: Collagen Type IX Alpha 2 Chain
CYB561: Cytochrome B561
DSC2: Desmocollin 2
EGF: Epidermal Growth Factor
EGFR: Epidermal Growth Factor Receptor
EGR1: Early Growth Response 1
ER: Estrogen Receptor
ESR1: Estrogen Receptor
FBP1: Fructose-Bisphosphatase 1
GATA3: GATA Binding Protein 3
GGT1: Gamma-Glutamyltransferase 1
GRB7: Growth Factor Receptor Bound Protein 7
GRO-seq: Global run on sequencing
H3K27me3: Histone 3, Lysine 27 Trimethyaltion
H3K4me3: Histone 3, Lysine 4 Trimethyaltion
HBEGF: Heparin Binding EGF Like Growth Factor
HDAC11: Histone Deacetylase 11
HER: Human Epidermal Growth Factor Receptor
HER2: Human Epidermal Growth Factor Receptor 2
HIF1AN: Hypoxia Inducible Factor Alpha Inhibitor
HLA-DOB: Major Histocompatibility Complex, Class II, DO Beta
HMCan: Histone Modifications In Cancer
IDE: Insulin Degrading Enzyme
ITPKC: Inositol-Trisphosphate 3-Kinase C
KEGG: Kyoto Encyclopedia of Genes and Genomes
KMO: Kynurenine 3-Monooxygenase
KRT7: Keratin 7
LIN7A: Lin-7 Homolog A, Crumbs Cell Polarity Complex Component
MACROD1: MACRO Domain Containing 1
MICALL2: MICAL-Like 2
MSMB: Microseminoprotein Beta
NR1H2: Nuclear Receptor Subfamily 1 Group H Member 2
PADI2: Peptidyl Arginine Deiminase 2
PAK5: Protein Activated Kinase 5
PCBP2: Poly (Rc) Binding Protein 2
PKD1: Polycystin 1, Transient Receptor Potential Channel Interacting
PRKACB: Protein Kinase Camp-Activated Catalytic Subunit Beta
RAB11A: RAB11A, Member RAS Oncogene Family
RNA: Ribonucleic Acid
RNF123: Ring Finger Protein 123
SHC4: SHC Adaptor Protein 4
SOX13: SRY-Box 13
SPRY4: Sprouty RTK Signaling Antagonist 4
SRA: Sequence Read Archive
ST3GAL6: ST3 Beta-Galactoside Alpha-2,3-Sialyltransferase 6
ST6GAL1: ST6 Beta-Galactoside Alpha-2,6-Sialyltransferase 1
STARD3: Star Related Lipid Transfer Domain Containing 3
TCGA: The Cancer Genome Atlas
TES: Testin LIM Domain Protein
TFF3: Trefoil Factor 3
TGFB3: Transforming Growth Factor Beta 3
TIMM17B: Translocase Of Inner Mitochondrial Membrane 17 Homolog B
TJP3: Tight Junction Protein 3
TMPRSS2: Transmembrane Protease, Serine 2
TSS: Transcription Start Site
TTLL4: Tubulin Tyrosine Ligase-Like 4
VLDLR: Very Low Density Lipoprotein Receptor
